# Manifestation of Incompleteness in Obsessive-Compulsive Disorder (OCD) as Reduced Functionality and Extended Activity beyond Task Completion

**DOI:** 10.1371/journal.pone.0025217

**Published:** 2011-09-23

**Authors:** Rama Zor, Henry Szechtman, Haggai Hermesh, Naomi A. Fineberg, David Eilam

**Affiliations:** 1 Department of Zoology, Tel-Aviv University, Ramat-Aviv, Israel; 2 Department of Psychiatry and Behavioural Neurosciences, McMaster University, Hamilton, Ontario, Canada; 3 Adult Outpatient Department and Anxiety Disorders and Behaviour Therapy Unit, Geha Mental Health Center, Rabin Medical Center, Petach-Tiquva, and Sackler Faculty of Medicine, Tel Aviv University, Ramat-Aviv, Israel; 4 National OCD Treatment Service, Hertfordshire Partnership NHS Foundation Trust, Queen Elizabeth II Hospital, Welwyn Garden City, United Kingdom; Freie Universitaet Berlin, Germany

## Abstract

**Background:**

This study focused on hypotheses regarding the source of incompleteness in obsessive-compulsive disorder (OCD). For this, we had to document the behavioral manifestation of incompleteness in compulsive rituals, predicting that an exaggerated focus on acts that are appropriate for the task will support the hypothesis on heightened responsibility/perfectionism. In contrast, activity past the expected terminal act for the motor task would support the “stop signal deficiency” hypothesis.

**Methodology and Principal Findings:**

We employed video-telemetry to analyze 39 motor OCD rituals and compared each with a similar task performed by a non-OCD individual, in order to objectively and explicitly determine the functional end of the activity. We found that 75% of OCD rituals comprised a “tail,” which is a section that follows the functional end of the task that the patients ascribed to their activity. The other 25% tailless rituals comprised a relatively high number and higher rate of repetition of non-functional acts. Thus, in rituals with tail, incompleteness was manifested by the mere presence of the tail whereas in tailless rituals, incompleteness was manifested by the reduced functionality of the task due to an inflated execution and repetition of non-functional acts.

**Conclusions:**

The prevalence of activity after the functional end (“tail”) and the elevated non-functionality in OCD motor rituals support the “lack of stop signal” theories as the underlying mechanism in OCD. Furthermore, the presence and content of the tail might have a therapeutic potential in cognitive-behavior therapy.

## Introduction

In their exposition of the conceptual issues underpinning biological psychiatry as a field of science, Berrios and Markova [1] noted that the discipline's evidence is dependent on two types of data: psychiatric data (psychopathology symptoms, signs, behaviors) and neurological data (neurology symptoms, signs, neuroimaging lesions, etc.). The authors indicated that the sophistication of neurological data was impressive but the collection and analysis of psychiatric data “*remains much the same as it was in the nineteenth century*” (p. 9). Specifically, what is unsatisfactory with current psychiatric data is the absence of behavioral descriptions of actual behaviors at a level of detail that is comparable with that of the physiological data.

As noted in the early years of OCD research, affective states are mapped in the flow of movements irrespective of whether the source for the mental state is emotional or physiological: …*"If psychological rumination' was all that there is, we should have nothing else to say; but the perturbation of intellect translates itself into acts… In a way, it is to be expected that obsessional ideas will become translated into empty acts, not adapted to reality"…* (Ribot, 1897; c.f. [2]). Accordingly, the analysis of observable behavior can serve as psychiatric data [3], [4], and this is obvious when the psychiatric disorder includes manifest behavior, for example, motor OCD rituals that serve as a source of information regarding mental symptoms. Analysis of such motor manifestation may shed light onto the various hypotheses regarding the psychological reasons for OCD symptoms [5].

In the present study, we analyzed OCD rituals in the context of two explanatory hypotheses for OCD symptoms. These hypotheses make distinct predictions as to the expected structure of rituals, and hence the outcome of behavioral analysis should provide evidence discounting one explanation. The two competing accounts considered here highlight different psychological attributes as reasons for OCD behavior. One asserts that OCD symptoms are produced by specific beliefs that pertain to responsibility and perfectionism, namely that one has a personal responsibility for protection against harm and that one should strive for perfection [6], [7], [8], [9], [10], [11], [12]. According to this type of account, those personally held beliefs could become exaggerated by producing an over-reaction to threat or uncertainty. This over-reaction, in turn, drives a corresponding increase in manifest responses to harm and uncertainty, observed as OCD symptoms. The second hypothesis asserts that OCD symptoms result from a dysfunction in the psychological process which marks that behavior has reached its intended end and ought to stop [13], [14]. This ‘stop’ process has a phenomenological counterpart, labeled variously as “a feeling of incompleteness” [15], [16], “just right feeling” [17], [18], or “a feeling of knowing” [14], [19]. According to this type of explanation, an individual who lacks the phenomenological experience of the stop signal continues to act and this performance perseveration manifests as OCD symptoms.

The heightened responsibility/perfectionism hypothesis predicts that the structure of OCD behavior is characterized by an exaggerated focus on acts that are the appropriate responses to threat and uncertainty, and hence a surplus of such actions. For instance, considering compulsive washing as an inflated response to the threat of contamination, the predicted structure of such washing rituals is a surfeit of hand massaging under the water and finally drying of hands, as these acts are directly relevant to the accomplishment of the intended function. In contrast, the ‘stop signal deficiency’ hypothesis predicts a continuation of motor activity past the expected terminal act - in the above example, past the drying of hands. To test these hypotheses, we employed video-telemetry of OCD rituals [3], [5], [20] to address a specific research question, namely, does the structure of OCD rituals provide evidence to differentiate between the above two competing theories of OCD symptoms. Applying video-telemetry in previous studies [3], [5], [20] revealed that motor rituals of OCD patients contained activity which was not necessary for the performed task (non-functional, or pessimal behavior) [3], [5], [20], and that this property helped to better distinguish between checking and cleaning rituals [3], [5], [20]. While previous studies with video-telemetry analyzed the entire content of motor performance, in the present study we utilized the definition of functional activity [3], [5], [20] to set a strict marker for the end for motor rituals, and thereby test whether motor behavior in OCD patients continues beyond this end point.

## Methods

### Subjects

Thirty OCD patients participated in this study. Nine patients were recruited from a British national specialist clinic serving a broad range of illness severity, including a high proportion of treatment-resistant cases. Twenty one patients were recruited from an Israeli regional psychiatric outpatient clinic and an anxiety disorders and cognitive behavioral therapy unit. All patients met DSM–IV criteria for OCD with insight [21], of at least a 12-month duration. In the very frequent case of comorbidity, OCD was the primary disorder, and none of the participants had demonstrated either Tourette's syndrome or a psychotic state. A previous study had revealed no cultural effect on OC behavior [22], thus the British and Israeli samples were pooled (see Table 1 for demographic details). Altogether, we studied 39 rituals, since nine of the 30 patients had two rituals. The content of these rituals was: 13 rituals of cleaning (the living room, sink, paint brush, dishes X 2, hands X 6, phone; wipe nose), four rituals of checking (stove, tap, kitchen, garage door), three rituals of preparing food (salad, tea, coffee), two rituals of brushing teeth, five rituals at door (locking x 4, opening), three rituals with a car (locking x 2, parking), three preparatory rituals (for going out from home x 2; before meal), and six other rituals (coffee break; make tea; organize cupboard; light a cigarette; fold towels; throw a cigarette packet). For each OCD ritual, a matched healthy individual of similar age, gender and nationality was selected, and requested to perform on camera the similar activity of the respective OCD patient [23]. The study was approved by the Helsinki Committee of the Geha Mental Health Center in Israel and the Hertfordshire Research Ethics Committee in the UK. After a complete description of the study to the subjects, written informed consent was obtained.

**Table 1:** Personal data of the 39 OCD patients.

Parameter	mean ± std
**Age** (years)	41±15
**Y-BOCS** (Total score)	26±8
**Y-BOCS** (Compulsions score)	14±4
**Age of OCD onset** (year)	19±12
**OCD duration** (years)	26±16

Prominent symptom dimension	Number of rituals
Checking/harming	19
Contamination/cleaning	10
Uncertain	10

### Procedure

Each participant was videotaped at her/his home, where, in the case of patients, the rituals were routinely performed. Y-BOCS severity scores were taken within the month prior to the video session. It was stipulated to the patients that she/he was requested to perform a current and frequently performed ritual. Control individuals were asked to perform the same task that formed the respective OCD ritual. For example, if a patient described a ritual as “cleaning the room”, the respective control was requested to “clean the room”. Videotaping commenced and lasted for 1-2 hours, with only the experimenter following the participant with a hand-held camcorder. The rituals did not seem to be affected by the presence of the experimenter since when asked after the session to rate the degree of similarity, patients reported a medium or higher degree of closeness of the videotaped ritual to their off-camera compulsion. Consistent with the patients' high ratings, we noted that once patients started to perform their rituals, performance took over and they paid no further attention to the observer or the camera but only to performing the ritual itself (see videoclip at http://www.tau.ac.il/lifesci/departments/zoology/members/eilam/eilam.html; note how the patient is concentrated in performance while the experimenter is nearby as evident by the reflection of her camcorder tripod in the mirror).

### Data acquisition and analysis

Motor behavior was scored during playback of the video files. We listed the multiple acts that comprised each compulsion, and this list served to score the behavior, using the Observer (Noldus Information Technology, the Netherlands), which is a software for behavioral descriptions. The beginning and end of a compulsion were determined by the patient's activity (see [20], [23] for details).

### Measures of functionality and other parameters of OCD behavior

#### Functional and non-functional acts

A comparison of the sequence of acts in a specific OC compulsion with the sequence of acts performed by the respective non-OCD control yielded two types of acts: (i) ***‘functional acts’*** that were performed by both the OCD patient and the respective non-OCD individual, and were therefore assumed to be necessary for task performance; (ii) ***‘non-functional acts’*** performed only by the OCD-patient or only by his/her respective control, and therefore assumed unnecessary for task performance [20], [23]. The rationale for the definition of non-functional acts was that the task could be completed without these acts. For example, an OCD patient performed a ‘brushing teeth’ ritual with the following sequence of acts: ‘put back tooth-brush’, ‘spit into the sink’, ‘spit towards the mirror’. The matching non-OCD individual also put back the tooth brush, spat into the sink but did not spit towards the mirror. Thus, the act ‘spit towards the mirror’ was classified as a non-functional act, performed only by the OCD patient. In general, a high rate of non-functional acts characterizes OCD behavior [20], [23].

#### Total number of all acts

The cumulative number of functional and non-functional acts (repetitions included).

#### Repertoire

The number of act types (repetitions excluded).

#### Rate of repetition

Calculated as the ‘total number of all acts’ divided by the repertoire. This parameter indicated the average rate of act repetition.

### Statistics

Unless noted differently, OCD patients and non-patients controls were compared using t-tests. Statistical analysis was performed using SPSS 15 for Windows and significance was set at P<0.05.

## Results

**Figure 1:**
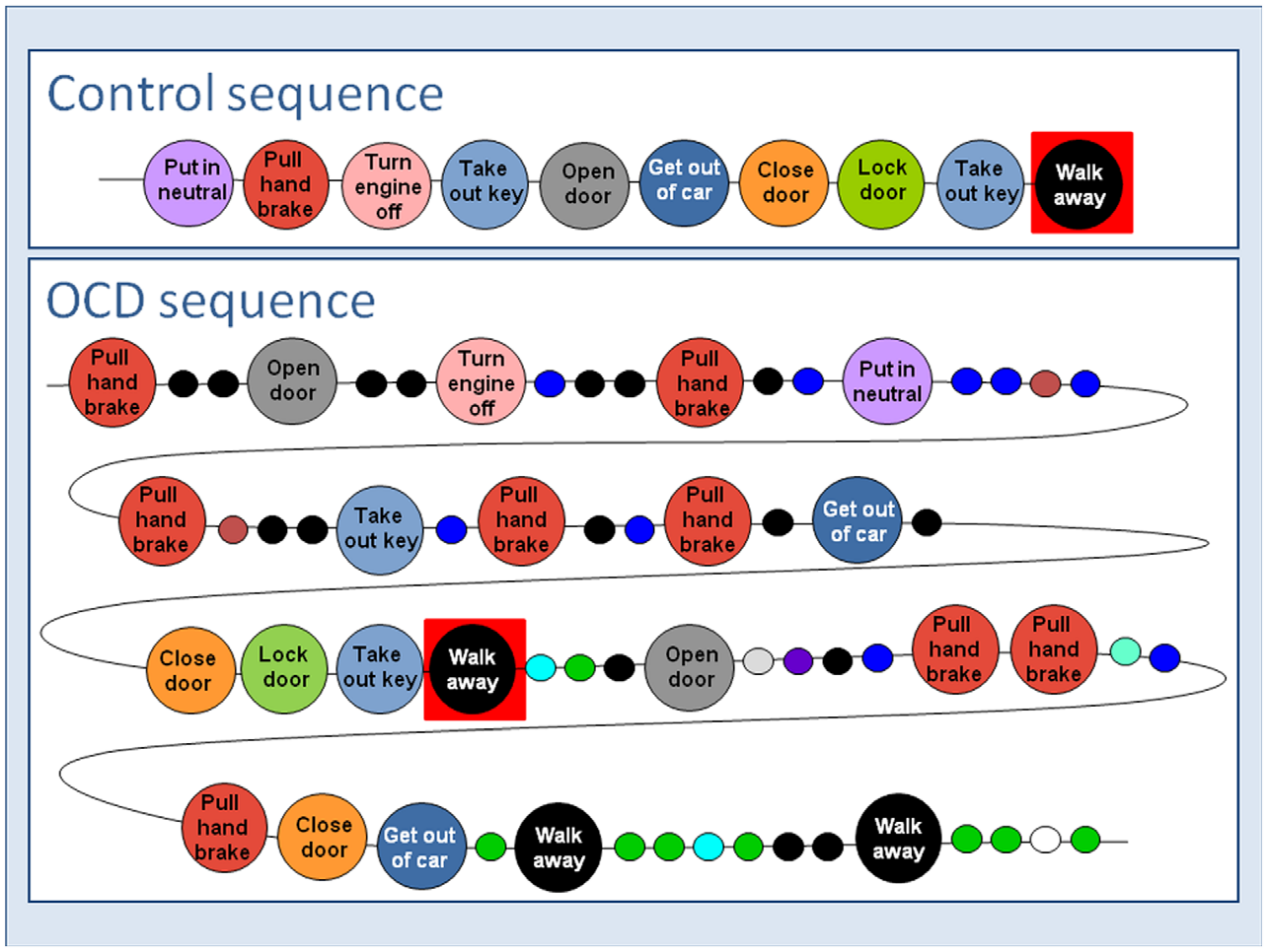
Sequences of functional acts (large circles) and non-functional acts (small circles).

The raw material for analysis was the actual sequence of acts performed in 39 OCD rituals. Figure 1 provides a graphic presentation of one such OCD ritual, ‘parking a car’ (bottom), and its corresponding non-OCD control behavior (top). Large circles represent acts that were shared in both the OCD and the control sequences; small circles represent acts that were unique to only the OCD or only the control sequence. The unique acts are considered ‘non-functional’ since the other individual could complete the task without these acts. Similarly, the shared acts are considered ‘functional’, being obligatory for the task. As shown, the OCD sequence is longer than the control (63 vs. 10 acts, respectively), comprising numerous non-functional acts (41 and 22 non-functional and functional acts respectively in the OCD sequence, compared with 10 and 0 such acts in the control sequence). In addition, the OCD sequence includes a higher rate of repetition for functional acts (2.2) and non-functional acts (5.86), whereas no act was repeated in the control sequence. Taken together, the sequence of functional acts in OCD was fragmented by the addition of multiple non-functional acts that broke the sequential order of the functional activity. In this sequence, we considered the first appearance of the last functional act (excluding repetitions) as the ***functional end of the task*** (depicted by a red square in both the OCD and the control sequences in Figure 1). However, the OCD ritual, unlike the control sequence, did not end at the ‘functional end’ (red square) as there were more acts after the red square before the ritual finally terminates. We refer to the sequence of acts from the beginning of the ritual to the functional end as ***‘task’***, and to the sequence of acts after the functional end as ***‘tail’***.

### Most OCD rituals contain a ‘tail’

**Figure 2:**
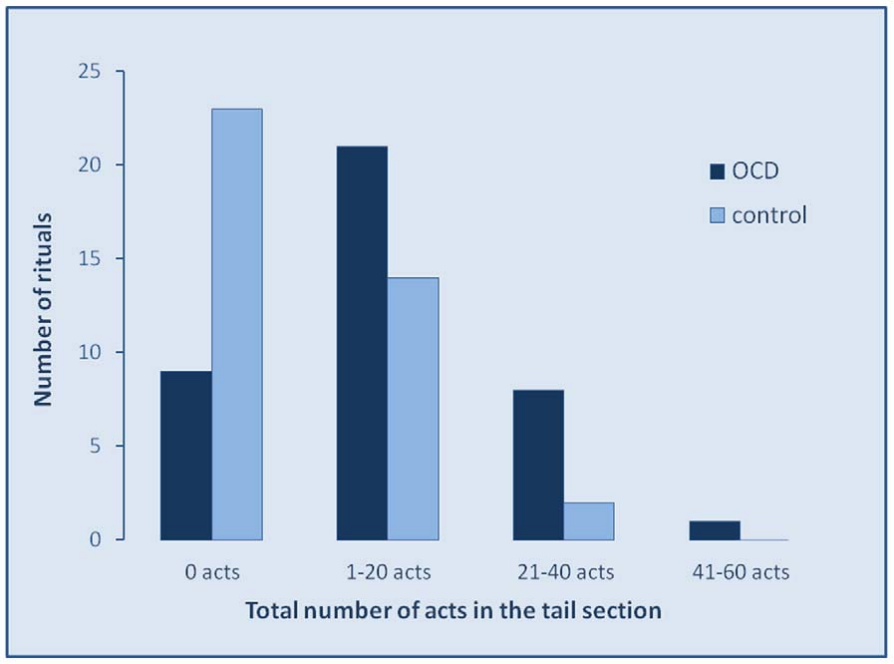
Frequency distribution of rituals according to the number of acts performed in the ‘tail’. As shown, most control sequences did not have a tail or had a tail with 1–20 acts. In contrast, only few (9) of the 39 OCD sequences did not have a tail, whereas the others had a tail with numerous acts (median of 12 acts compared with a median of 2 acts in control sequences).

A frequency distribution of the acts performed past the ‘functional end’ of the ritual, as observed in 39 OCD rituals and their 39 respective control sequences (Figure 2) revealed that most control sequences (23/39) did not continue beyond the functional end, but a minority did (16/39), with 1 to 30 acts past the task (median of 2 acts). In contrast, most OCD rituals (30/39) had a tail of 1 to 60 acts (median of 12 acts). The proportion of sequences with a tail was significantly higher in OCD than in control sequences (77% and 41% in OCD and control sequences, respectively; χ^2^ = 10.38, p = 0.001).

### The ‘tail’ of OCD rituals is long and contains mostly non-functional acts

**Figure 3:**
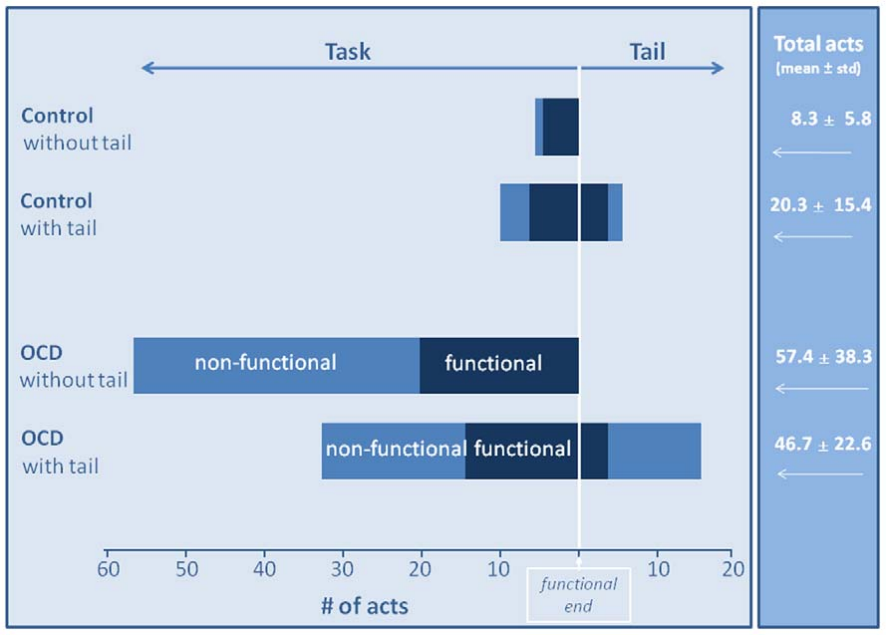
Functional acts (dark blue) and non-functional acts (light blue) in the task and tail sections.

Figure 3 displays as a stacked bar graph the mean number of functional and non-functional acts in rituals with and without tail. As shown, tail length and composition differed between control and OCD rituals. The tail was significantly longer in OCD rituals, with 14.6±2.6 acts compared to 5.7±2.3 acts in the tail of control sequences (t_44_  = 2.03, P = 0.026). Furthermore, tail composition was dominated by non-functional acts in OCD rituals (3.1±0.7 and 11.7±2.1 functional and non-functional acts, respectively, t_29_ = 4.803, P<0.001, paired t-test) but not in the control sequences (3.4±1.3 and 2.4±1.0 functional and non-functional acts, respectively; t_15_ = 1.754, P = 0.100, paired t-test). Taken together, the data show an expansion of OCD performance beyond the normal task endpoint by addition of mainly non-functional acts.

### The ‘tail’ influences performance of OCD and control rituals differently

As noted, not all OCD rituals had a tail and not every control sequence was without one. Inspection of Figure 3 suggests that the tail had a different influence on performance of control and OCD sequences in the following three ways.

First, the tail increased the total number of acts in control but not in OCD sequences: In control sequences without a tail there were 8.3±1.2 acts compared to 20.3±3.8 acts in sequences with a tail (t_37_ = 3.438, P = 0.001). In contrast, in OCD sequences, the total number of acts remained the same, regardless of the tail (57.4±12.8 acts in OCD sequences without a tail compared with 46.7±4.1 acts in OCD sequences with tail; t_37_ = 1.059, P = 0.296).

Second, the tail elevated performance of non-functional acts in the task of control sequences but reduced their frequency in the task of OCD rituals. Specifically, the number of non-functional acts in the task section of control sequences with tail was significantly higher than in the task of sequences without tail (4.8+1.7 acts and 1.0+0.4 acts, respectively; t_37_ = 2.553, P = 0.015). In contrast, in OCD rituals, the number of non-functional acts in the task section decreased from 37.1+11.9 acts in OCD sequences without a tail to 17.8+3.1 acts in OCD sequences with a tail (t_37_ = 2.267, P = 0.029).

Third, the rate of repetition of non-functional acts was significantly higher in OCD sequences without tail compared with OCD sequences with tail (3.50±1.60 vs. 2.07±1.08, respectively, t_10_ = 2.52, P = 0.03). The rate of repetition of functional acts did not differ in OCD sequences with or without tail, and did not differ for functional and non-functional acts in controls sequences with or without tail (data not shown).

In all, these data suggest that a different process may underlie the appearance of the tail in control and OCD sequences. It appears that the tail in OCD rituals may reflect a shift of non-functional performance into it, at the expense of the task section. In other words, OCD sequences either include a tail that is mostly non-functional, or comprise a higher rate of repetition on non-functional acts in sequences without a tail. In contrast, the tail in control sequences may reflect a small extension of performance beyond the functional endpoint (Figure 3).

## Discussion

We found here that the majority of OCD rituals (75%) comprised a ‘**tail**’, which is a section that follows the functional end of the task that the patients ascribed to their activity. That is, OCD patients typically continued their activity beyond the functional (pragmatic) end of the task. Moreover, acts in the tail were mostly non-functional, and the task could be completed without these acts. Specifically, compared with non-OCD controls, significantly more OCD rituals comprised a ‘tail’. Furthermore, OCD ‘tails’ were longer in duration and in the number of acts, comprising significantly more *non-functional* acts (see Figure 1 and Figure 3). It should be emphasized that the inclusion of the tail in the analyzed behavior was not a decision of the observer, but a decision of the OCD and non-OCD individuals, who spontaneously included the tail in their performance and terminated their activity beyond the ‘functional end’. Since a lesser version of the ‘tail’ occurred in non-OCD individuals, it seems that the ‘tail’ is a normal and intrinsic continuation of task fulfillment, and that this normal part is inflated in OCD. Indeed, pathological symptoms in OCD are known as an exaggeration of normal components (DSM-IV, [21]). However, non-OCD individuals tend to minimize their sequence of acts and perform mainly necessary (functional) acts, whereas in OCD the longer tail is mainly non-functional. It should be noted, however, that 25% of OCD rituals in our sample were without a tail. Accordingly, we suggest that OCD motor rituals dichotomize into two types that reflect two behavioral notions on incompleteness: (i) rituals with relatively higher functionality in the task section, followed by a highly non-functional tail; and (ii) rituals without tail that comprise a relatively high number and higher rate of repetition of non-functional acts. The present notion on non-functionality in OCD rituals accords with a recent study in which OCD patients who were trained to accomplish a computer task were prone to slips-of-action, and this suggests that they had a deficit in controlling goal-directed tasks [24]. We propose that in rituals with a tail, incompleteness is manifested by the mere presence of the tail, whereas in rituals without a tail, incompleteness is manifested by the reduced functionality of the task due to the inflated execution and repetition of non-functional acts. In the following, we discuss the possible cognitive mechanisms suggested to underlie incompleteness in OCD, which according to our view may explain the differences between the two behavioral notions as ascribed above.

‘Perfectionism’ was suggested to be driven by the *‘attempt to maintain control by reducing the risk of harm and insuring safety*’ [25]. Perfectionists trust that they can and should reach perfect performance, and therefore regard anything less than perfect performance as unsatisfactory [26]. OCD significantly correlates with perfectionism characteristics (dimensions) as defined by the Frost Multidimensional Perfectionism Scale (MPS-F). Specifically, OCD is associated with ‘doubts about actions’ and ‘concern over mistakes’ dimensions [25], [26], [27]. Other cognitive mechanisms include ‘put matters right’ [28] and the ‘not just right experience’ [10], [28], [29], [30], [31], [32], [33]. The NJRE feeling of OCD patients was hypothesized to serve as an inner indicator or evidence in the decision that the task was completed [18]. Both ‘perfectionism’ and ‘NJRE’ mechanisms relate to the persistence and rigidity that characterizes OCD symptoms [21]. OCD patients may be extremely motivated to reach some specific performance goal, the ambition of which is to obtain an outcome consistent with their own standards. Accordingly, OCD patients who attempt to achieve a ‘perfect’ or ‘just right’ performance would be expected to perform a large core of functional activity that is necessary for task completion. In the context of the present study, these patients should perform more functional acts, especially in the tail, in order to ensure that the task would be properly completed. However, our present results clearly demonstrate that the excessive activity in OCD patients comprises mainly *non-functional* acts (see also [20]). Additionally, the inspection of only the ‘tail’ section revealed more *non-functional* than *functional* activity compared with non-OCD tails. Thus, the behavioral manifestation of incompleteness either as a 'tail' or as a high frequency of non-functional acts (or both) implies that mechanisms such as ‘perfectionism’, ‘NJRE’, etc., are not the appropriate explanation for incompleteness in OCD.

On the other hand, cognitive mechanisms that focus on *non-functional* characteristics seem to adhere with the present findings on OCD behavior. Specifically, the ‘precautionary system’ [34] and ‘security motivation’ [35], [36] are cognitive mechanisms that interpret the excessive activity in OCD rituals as neutralizing behavior, performed in response to a potential harm. These mechanisms support the notion that OCD patients suffer from a lack of a ‘stop’ signal or criterion, thereby continuing to act even after task-completion. More specifically, patients with OCD are thought to experience an inflated sense of responsibility that derives from an exaggerated evaluation of unimportant situations as dangerous, resulting in excessive neutralizing behavior aimed at harm avoidance [10], [11], [28], [37], [38]. According to this theory, ‘responsibility’ generates a problem in decision making, with OCD patients requiring more evidence to decide that a task has been completed [18]. The required evidence is external such as the sound of the door being locked, and subjective such as the ‘feeling’ that the door is locked properly. In OCD, the subjective feeling of having completed an action malfunctions, i.e. the stop-signal fails, and therefore the ability to terminate the task is damaged. In the same vein, ‘security motivation’ [35], [36] and the ‘precautionary system’ [34] were theorized as a complementary mechanism for encouraging neutralizing behaviors since the prevention of the occurrence of a potential threat that does not in actuality occur and therefore does not have an external terminating signal, may additionally contribute to the inability to stop acting. The resultant prolonged activity does not need to be necessarily linked to the task, and accordingly may be mediated by non-functional activity. In other words, the failure of the internal ‘stop-signal’ may generate an unpleasant feeling of incompleteness that results in the needless and excessive repetition of acts. These acts do not need to be ‘functional’ as they are simply designed to bring about a sense of relief and reduce the generally unpleasant feeling of incompleteness.

Altogether, the present study provides evidence for the behavioral manifestation of incompleteness in motor behavior of OCD patients. Focusing on motor behavior limits the present findings to overt compulsions, and they do not necessarily explain the experience of repugnant obsessions that is quite common in OCD. Utilizing video-telemetry we demonstrate that 75% OCD rituals comprise a ‘tail’ that follows the completion of the task. While a tail may also occur in non-OCD behavior, in OCD it is inflated in duration and in number of acts, which are mostly non-functional. Since this activity occurs after the task is practically completed, it appears to represent a manifestation of incompleteness. The 25% of tailless OCD rituals feature reduced functionality and high repetitiveness, which are also a behavioral manifestation of incompleteness. In all, the elevated non-functionality is consistent with the concepts of security motivation, precaution system, and other ‘lack of stop signal theories’ as one of the underlying mechanisms in OCD. The present behavioral evidence for incompleteness should be further implemented in attempts to link between cognitive theories on the underlying mechanism and the phenotypes of OCD. Moreover, it might be translated to novel therapeutic steps in cognitive- behavior therapy of OCD patients.
